# Supplementary material for: Identification of Biomarkers Co-Associated with Lactylation and Acetylation in Systemic Lupus Erythematosus
Source: Biomedicines. 2025 May 22;13(6):1274. doi: 10.3390/biomedicines13061274 (PMC12189252; doi:10.3390/biomedicines13061274)
Supplement: Supplementary file 1 [file biomedicines-13-01274-s001.zip › Supplementary Material S1-Clinical manifestation and laboratory data of SLE patients and normal controls.pdf]

Demographic and Clinical Characteristics of Study Participants

Parameter	Healthy controls (n=10)	SLE Patients (n = 14)	Statistical Test	p-value	Adjusted in Analysis
Demographics					
Age (mean + SD)	29 ± 6	39±17	Kolmogorov -Smirnov	0.12	ANCOVA
Sex (M/F)	3/7	3/11	Fisher's exact test	0.7	-
SLEDAI	-	10±6	-	-	-
Metabolic Parameters					
BMI (kg/m²)	22.3 ± 2.1	23.7 ± 3.8	Independent t-test	0.27	ANCOVA
Systolic BP (mmHg)	118 ± 10	124 ± 15	Mann-Whitney U-test	0.21	-
Fasting Glucose (mmol/L)	4.8 ± 0.5	5.1 ± 0.7	Independent t-test	0.12	-
2019 ACR/EULAR Classification Criteria for SLE					
Malar rash	-	10	-	-	-
Discoid rash	-	3	-	-	-
Photosensitivity	-	8	-	-	-
Oral ulcers	-	2	-	-	-
Nonerosive arthritis	-	6	-	-	-
Pleuritis or pericarditis	-	1	-	-	-
Nephritis	-	4	-	-	-
Neurological disorder	-	1	-	-	-
Hematological disorder	-	1	-	-	-
Immunological disorder	-	14	-	-	-
Positive antinuclear antibody (ANA)	-	14	-	-	-

Positive Anti-dsDNA antibody (dsDNA)	-	13	-	-	-
WBC (×10^9/L)[a]	6.2 ± 1.5	4.70±2.07	Mann-Whitney U-test	0.045	-
Neutrophils (×10^9/L)[b]	3.5 ± 1.0	4.78 ± 0.31	Welch's t-test	0.15	-
Lymphocytes (×10^9/L)[c]	2.0 ± 0.6	1.37 ± 0.22	Independent t-test	<0.001	-
Platelets (×10^9/L)[d]	250 ± 45	187.00±73.00	Welch's t-test	0.005	-
C3 (g/dL)	1.2 ± 0.3	0.70±0.34	Mann-Whitney U-test	<0.001	-
C4 (g/dL)	1.2 ± 0.3	0.12±0.09	Mann-Whitney U-test	<0.001	-

SLE Systemic Lupus Erythematosus, SLEDAI SLE Disease Activity Index, BP Blood Pressure, ACR American College of Rheumatology, EULAR European League Against Rheumatism, C3 Complement component 3, C4 Complement component 4

[a]Normal range of white blood cell (WBC) count: 3.5–10.5 × 10^9/L

[b]Normal range of neutrophils: 2.0–7.0 × 10^9/L

[c]Normal range of lymphocytes: 1.0–3.0 × 10^9/L

[d]Normal range of platelets: 125-350× 10^9/L
