# Supplementary material for: Identification of Biomarkers Co-Associated with Lactylation and Acetylation in Systemic Lupus Erythematosus
Source: Biomedicines. 2025 May 22;13(6):1274. doi: 10.3390/biomedicines13061274 (PMC12189252; doi:10.3390/biomedicines13061274)
Supplement: Supplementary file 1 [file biomedicines-13-01274-s001.zip › Supplementary Material S2.pdf]

Supplementary Material S2

qRT-PCR Validation of OXNAD1 and H1-2 Expression

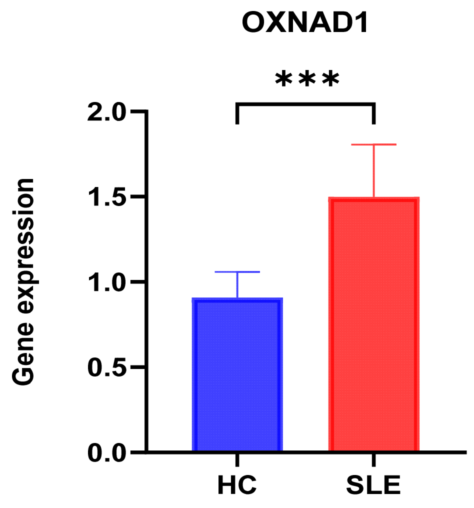

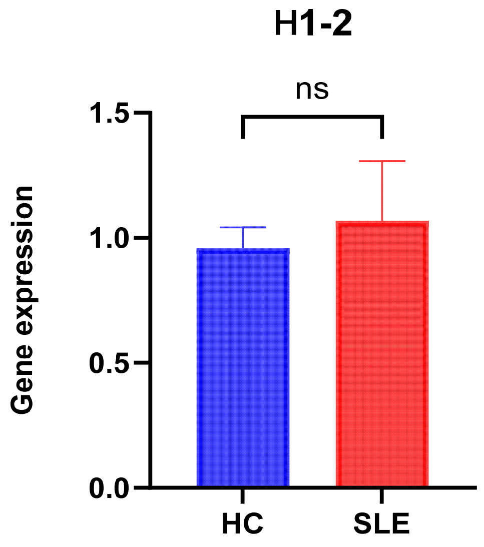

Relative mRNA expression levels of OXNAD1 and H1-2 were measured by qRT-PCR in PBMCs from SLE patients (n = 14) and healthy controls (n = 10). Gene expression was normalized to GAPDH. Statistical significance is indicated as follows: *P < 0.05, **P < 0.01, ***P < 0.001, ****P < 0.0001, ns, no significance.
